# Predictive value of the RIFLE urine output criteria on contrast-induced nephropathy in critically ill patients

**DOI:** 10.1186/s12882-016-0243-5

**Published:** 2016-03-28

**Authors:** Aldjia Hocine, Pierre Defrance, Jacques Lalmand, Christian Delcour, Patrick Biston, Michaël Piagnerelli

**Affiliations:** Intensive Care, CHU-Charleroi, Université Libre de Bruxelles, 6042 Charleroi, Belgium; Cardiology, CHU-Charleroi, Université Libre de Bruxelles, 6042 Charleroi, Belgium; Radiology, CHU-Charleroi, Université Libre de Bruxelles, 6042 Charleroi, Belgium; Experimental Medicine Laboratory, Université Libre de Bruxelles 222 Unit, CHU-Charleroi, 6111 Charleroi, Belgium

**Keywords:** Renal failure, Critically ill, Contrast-induced nephropathy, RIFLE

## Abstract

**Background:**

To investigate the predictive value of decreased urine output based on the Risk of renal dysfunction, Injury to the kidney, Failure of kidney function, Loss of kidney function and End-stage renal disease (RIFLE) classification on contrast- induced acute kidney injury (CA-AKI) in intensive care (ICU) patients.

**Methods:**

All patients who received contrast media (CM) injection for CT scan or coronary angiography during a 3-year period in a 24 bed medico-surgical ICU were reviewed.

**Results:**

Daily serum creatinine concentrations and diuresis were measured for 3 days after CM injection. We identified 23 cases of CA-AKI in the 149 patients included (15.4 %). Patients who developed CA-AKI were more likely to require renal replacement therapy and had higher ICU mortality rates. At least one RIFLE urine output criteria was observed in 45 patients (30.2 %) and 14 of these 45 patients (31.1 %) developed CA-AKI based on creatinine concentrations. In 30 % of these cases, urine output decreased or didn’t change after the increase in creatinine concentrations. The RIFLE urine output criteria had low sensitivity (39.1 %) and specificity (67.9 %) for prediction of CA-AKI, a low positive predictive value of 50 % and a negative predictive value of 87.2 %. The maximal dose of vasopressors before CM was the only independent predictive factor for CA-AKI.

**Conclusions:**

CA-AKI is a frequent pathology observed in ICU patients and is associated with increased need for renal replacement therapy and increased mortality. The predictive value of RIFLE urine output criteria for the development of CA-AKI based on creatinine concentrations was low, which limits its use for assessing the effects of therapeutic interventions on the development and progression of AKI.

## Background

Acute kidney injury (AKI) is a frequent pathology in critically ill patients: a European epidemiological survey reported that 25 % of patients had transient AKI and that 10 % of them needed renal replacement therapy (RRT) during their intensive care (ICU) stay [1, 2]. The mortality rate associated with AKI ranges from 30 % in toxic forms to 90 % when associated with multiorgan failure [1, 3].

The physiopathology of AKI is multifactorial in critically ill patients and include low systemic blood pressure [4–6], intravascular hypovolaemia [7], alterations of the local microcirculation [7, 8], systemic inflammation with renal leukocyte accumulation [9], ischaemia/reperfusion processes [10], and direct drug toxicity [11].

Contrast-induced nephropathy is a common cause of hospital-acquired AKI [12]. The incidence of this condition varies across studies but it appears to be much higher in ICU patients [13], varying from 2 to 23 % in a recent retrospective monocenter study [14, 15].

The pathophysiology of contrast-induced nephropathy is complex and associates vasoconstriction with renal ischaemia, oxidative stresses, inflammation and direct toxicity of the iodinated contrast media (CM) on tubular cells, leading finally to an increase in serum creatinine [16, 17]. Identification of contrast-induced nephropathy in ICU patients is difficult because of the many other pathological mechanisms complicating critical illness. For these reasons, Hoste et al. [15] suggested using the term contrast-associated AKI (CA-AKI) instead of “induced”. CA-AKI is defined by an increase in serum creatinine of 25 % from baseline or ≥0.5 mg/dL within 3 days after CM administration [18]. Because of the high mortality rate associated with AKI in critically ill patients, early detection of CA-AKI may be important to enable therapy to be adapted accordingly. To provide a uniformly accepted definition of AKI, a classification associating change in urine output and increase in serum creatinine has been proposed: the RIFLE (Risk of renal dysfunction, Injury to the kidney, Failure of kidney function, Loss of kidney function and End-stage renal disease) classification [19, 20]. This classification shows the better robustness and a higher detection rate during the first 48 h of ICU admission in several studies [20–22]. More recently, the Kidney Disease Improving Global Outcome (KDIGO) criteria, an alternative to the RIFLE criteria was developed but short-term and long-term mortality were similar.

Because intermittent oliguria may turn into persistent oliguria or evolve into AKI [23, 24] and because AKI with oliguria or anuria has been reported to be associated with a worse outcome compared with patients with preserved urine output and because urinary output is easy to measure with indwelling urinary catheters in ICU patients, we hypothesised that the RIFLE urine output criteria could be an early and predictive marker of CA-AKI. We, therefore, evaluated the incidence of CA-AKI and the RIFLE urine output criteria in all patients who received CM in our ICU over a 3-year period.

## Methods

### Patients

The local ethical committee (ethical committee from the Intercommunal de Santé Publique du Pays de Charleroi-OM008) approved this study and informed consent was waived because of the retrospective nature that required no intervention. We reviewed the data from all patients admitted to our 24-bed medico-surgical ICU from 1st January 2010 to 31st December 2012 who received intravenous or intra-arterial CM injection for computed tomography (CT) or coronary angiography.

We included all patients ≥18 years, with an ICU length stay of minimum 3 days after the CM injection. Exclusion criteria were: renal transplantation, decision to start RRT before CM injection, end-stage renal disease requiring iterative haemodialysis, another injection of CM 3 days before or after the analysed examination, surgery within 3 days after the CM injection, incomplete biological or demographic data.

We recorded the age, sex, weight, diagnosis at ICU admission (cardiological, including cardiac arrest, myocardial infarction and cardiogenic shock; trauma; sepsis; suspicion of pulmonary embolism; neurologic, including subarachnoid or brain haemorrhages or brain tumor; or other (renal imaging, suspicion of mesenteric ischaemia, etc.). We also collected the Acute Physiology And Chronic Health Evaluation (APACHE) II score [25], co-morbidities known to alter renal function, e.g., diabetes mellitus, cardiovascular risk factors including ischaemic cardiomyopathy and/or arterial hypertension, and prior chronic renal failure defined by a creatinine concentration ≥1.5 mg/dL [15]. We also recorded the need for RRT during the ICU hospitalisation because of classical complications of renal failure, i.e., overload, acidosis, hyperkalaemia or hyperuremia [26], the length of ICU and hospital stays and the ICU mortality.

Any treatments that can be involved in the development of AKI were also noted, including diuretics, non-steroidal anti-inflammatory drugs, angiotensin-converting enzyme inhibitors or angiotensin II receptor blockers, aminoglycosides and glycopeptides. Maximum doses of vasoactive drugs (norepinephrine and dobutamine) before and every day after CM administration were recorded.

Any specific preventive therapy given before the CM was also noted: e.g., administration of N-acetylcysteine or specific fluid administration. However, no protocol for CA-AKI prevention was applied routinely in our ICU during the study period. The CM used during the study period were all non-ionic, and iso-osmolar or low-osmolar. Angiography examinations were exclusively performed with a non-ionic, iso-osmolar contrast agent. We also recorded the quantity of CM injected by body weight.

### Biological data

We retrieved the biological data that was closest the injection of CM and at days 1, 2 and 3 after injection. These data included: haematocrit, blood urea, creatinine (Abbott Laboratories, Illinois, USA; coefficient of variation :0.6 % or 0.1 mg/dL), bicarbonate, natremia, C-reactive protein, and creatinine phosphokinase concentrations. Glomerular filtration rate was estimated as the measured and estimated glomerular filtration rate (MDRD) [27]. We also collected, at the same time, blood lactate concentrations and blood gases including pH, and base excess.

### Urine output and haemodynamic data

We reviewed the haemodynamic data before, and 6, 12, and 24 h after CM injection and on days 2 and 3. At these times, we recorded the mean arterial blood pressure and central venous pressure. For each day, we calculated the quantity of liquids administered (oral and intravenous). Because hourly diuresis being followed in ICU patients, we summed the amount of urine output each 6 h post CM injection.

### Diagnosis of CA-AKI

We identified cases of CA-AKI according to the definition proposed by Barrett and Parfrey [18]: increase in serum creatinine by 25 % from baseline or a minimum of 0.5 mg/dL within 3 days after CM administration. The baseline creatinine was that measured on the day of the injection.

We classified patients in relation to the RIFLE urine output criteria [19]: -R (Risk): < 0.5 ml/kg/h for 6 h,- I (Injury): < 0.5 ml/kg/h for 12 h -F (Failure): < 0.3 ml/kg/h for 24 h or anuria for 12 h.

### Statistical analysis

Data are presented as median values with [25–75th] percentiles or number (%) as appropriate. We used Mann–Whitney or Kruskal-Wallis one way analysis of variance for comparisons between groups. Changes over time in creatinine concentrations, urine output, mean blood pressure and fluid intake were analyzed using a Friedman repeated measures analysis. A *p* value <0.05 was considered as significant. To identify potential risk factors for developing CA-AKI based on the definition proposed by Barrett and Parfrey [18] or death, we performed a logistic regression analysis including all variables with a difference at the significance level ≤0.2 between patients who did and did not develop CA-AKI. Because of the largely non Gaussian distribution, the square root value of the doses of vasopressors dose was introduced in the logistic regression model. In cases in which two covariates were highly correlated, only one of the two covariates was included in the model. Results are reported as odds ratios (ORs) with 95 % confidence intervals.

Statistical analyses were performed using the SigmaStat v 12.0 software package (Systat Software Inc, San Jose, Cal) and logistic regression analysis was performed using XLSTAT 2013 (Addinsoft).

## Results

### Patients

**Fig. 1:**
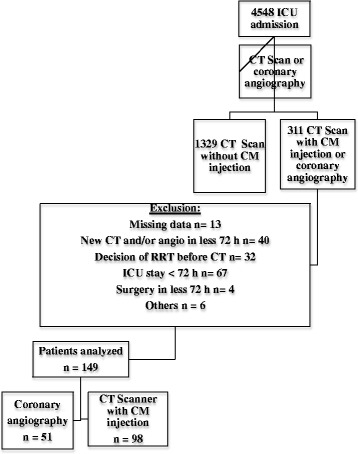
Flow chart of the patients included in the study

During the 3-year study period, 4548 patients were admitted to our ICU. Thousand six hundred forty patients had a CT of whom 311 had a CT with CM or a coronary angiography. After exclusion of 162 patients (Fig. 1), the final analysis included 149 patients (98 CT and 51 coronary angiography).

The patients’ characteristics are summarized in Table 1. Their median age was 64 [56–72] years, 62 % were male, and the median APACHE II score was 20 [14–25]. Twenty eight percent had diabetes and 70 % had cardiovascular risk factors. The majority of the admissions were for medical reasons (75.8 %). The median ICU length of stay was 12 [7–21] days and ICU mortality was 35 %.

**Table 1:** Clinical, biological and haemodynamic characteristics of the total population (*n* = 149) before CM injection

Age (years)	64 [56–72]
Sex (male, %)	92 (62)
ICU admission diagnosis (medical/surgical)	113/36
APACHE II score	20 [14–25]
Weight (kg)	78.0 [70.0–90.0]
Diabetes mellitus (%)	41 (27.5)
Cardiovascular risk factors (%)	104 (69.8)
Need for renal replacement (%)	6 (4)
Length of ICU stay (days)	12 [7–21]
ICU mortality (%)	35 (23.5)
Haematocrit (%)	31.4 [27.1–38.3]
Urea (mg/dL)	44.4 [32.0–58.1]
Creatinine (mg/dL)	0.91 [0.64–1.13]
MDRD (mL/min/1.73 m^2^)	77.7 [54.5–113.4]
Patients with creatinine ≥1.5 mg/dL (%)	15 (10)
pH	7.40 [7.33–7.46]
BE (meq/L)	0.00 [−3.00–4.25]
HCO3 (mg/dL)	24.8 [21.6–29.2]
Na (mEq/L)	140 ± 5
Lactate (mmol/L)	0.5 [0.4–0.8]
Nephropathy prevention (%)	29 (19.5)
Mean blood pressure before CM injection (mmHg)	81 [71–93]
CVP (mmHg)	9 [6–12]
Maximal vasopressor dose (mcg/kg/min)	0.00 [0.00–0.08]
Fluid intake, day 0 (mL)	2830 [2114–3621]
Doses of CM (ml/kg)	1.6 [1.31–2.29]
Nephrotoxic drugs (%)	66 (44.3)
Diuretics (%)	24 (16.1)

*BE* base excess

### Diagnosis of CA-AKI

**Fig. 2:**
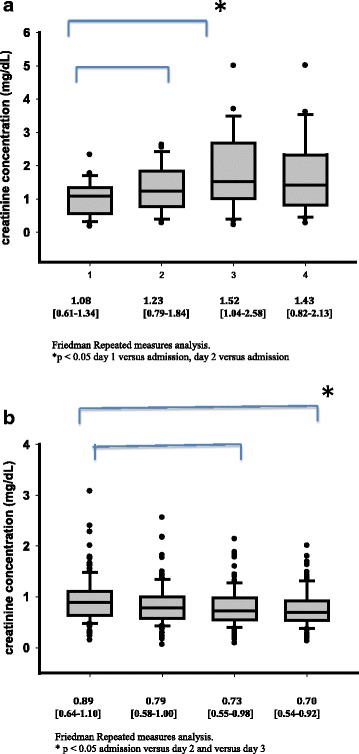
Time course of serum creatinine concentrations in patients who developed CA-AKI (**a**) and those who did not (**b**). Friedman Repeated Measures Analysis

There were 23 cases of CA-AKI (15.4 %) in our cohort, based on an increase in serum creatinine from 1.08 [0.61–1.34] mg/dL before CM injection to 1.43 [0.82–2.13] mg/dL at day 3 (*p* < 0.001) (Fig. 2a). In contrast, creatinine concentrations decreased overtime in patients without CA-AKI (from 0.89 [0.64–1.10] to 0.70 [0.54–0.92] mg/dL, *p* < 0.05; Fig. 2b). Thirteen of these cases were in patients who had a CT scan (13.3 % of all patients who underwent a CT with CM) and ten in patients who had undergone coronary angiography (19.7 % of all patients who underwent a coronary angiography). Comparisons of clinical characteristics and biological data in patients who developed CA-AKI and those who did not are shown in Table 2. At the time of CM injection, urea and serum creatinine concentrations were comparable in the two groups. More patients who developed CA-AKI needed RRT (13 vs 2 %, *p* = 0.02) and CA-AKI was associated with higher ICU mortality (52 versus 19 %, *p* < 0.001). Although mean blood pressure remained stable during the study period in patients who developed CA-AKI, values were significantly lower already after 6 h post CM injection and during the study period compared to patients who did not develop CA-AKI (Table 2).

**Table 2:** Comparisons of clinical, biological and haemodynamic characteristics between patients who developed or not CA-AKI

	CA-AKI (*n* = 23)	No CA-AKI (*n* = 126)	*P* value
Age (years)	65 [55–78]	64 [56–72]	0.43
Sex (male/female)	16/7	76/50	0.41
ICU admission diagnosis (medical/surgical)	20/3	93/33	0.18
APACHE II score	25 [18–30]	19 [14–24]	0.006
Weight (kg)	80 [70–90]	78 [70–90]	0.91
Diabetes mellitus (%)	6 (26)	35 (28)	0.87
Cardiovascular risk factors (%)	17 (74)	87 (69)	0.64
Need for renal replacement (%)	3 (13)	3 (2)	0.02
Length of ICU stay (days)	13 [6–18]	12 [7–22]	0.99
ICU mortality (%)	12 (52)	23 (19)	<0.001
Haematocrit (%)	31.5 [27.3–33.5]	31.4 [27.0–38.2]	0.78
Urea (mg/dL)	47.9 [35.2–56.4]	44.1 [31.8–58.5]	0.63
Creatinine (mg/dL)	1.08 [0.61–1.34]	0.89 [0.64–1.10]	0.26
MDRD (mL/min/1.73 m^2^)	61.0 [49.4–105.0]	79.4 [57.4–116.0]	0.15
Patients with creatinine ≥1.5 mg/dL (%)	3 (13 %)	12 (9.5 %)	0.61
pH	7.37 [7.28–7.47]	7.40 [7.33–7.46]	0.39
BE (meq/L)	−1.5 [−4.0 − 3.0]	0.0 [−3.0–4.0]	0.38
HCO3 (mg/dL)	23.6 [20.7–31.3]	25.3 [23.0–29.3]	0.54
Na (mEq/L)	138 [134–142]	139 [137–143]	0.14
Lactate (mmol/L)	0.7 [0.5–1.3]	0.5 [0.4–0.8]	0.02
Nephropathy prevention (%)	3 (13)	26 (20)	0.40
Mean blood pressure before CM (mmHg)	79 [62–89]	81 [72–93]	0.15
Mean blood pressure at 6 h post CM injection (mmHg)	73 [67–82]	81 [70–94]	0.056
Mean blood pressure at 12 h post CM injection (mmHg)	73 [66–76]	77 [71–88]	0.012
Mean blood pressure at 24 h post CM injection (mmHg)	78 [71–87]	79 [70–94]	0.092
Mean blood pressure at 48 h post CM injection (mmHg)	75 [68–90]	85 [77–98]	0.001
Mean blood pressure at 72 h post CM injection (mmHg)	76 [70–87]	85 [75–100]	0.008
CVP before CM (mmHg)	13 [8–14]	9 [6–11]	0.12
Maximal vasopressor dose before CM (mcg/kg/min)	0.07 [0.00–0.20]	0.00 [0.00–0.05]	0.001
Maximal vasopressor dose day 1 (mcg/kg/min)	0.07 [0.00–0.26]	0.00 [0.00–0.00]	<0.001
Maximal vasopressor dose day 2 (mcg/kg/min)	0.04 [0.00–0.35]	0.00 [0.00–0.00]	<0.001
Maximal vasopressor dose day 3 (mcg/kg/min)	0.03 [0.00–0.29]	0.00 [0.00–0.00]	<0.001
Doses of CM (ml/kg)	1.71 [1.44–3.47]	1.60 [1.30–2.00]	0. 14
Nephrotoxic drugs (%)	9 (39.1)	57 (46.3)	0.59
Diuretics on day of CM injection (%)	5 (21.7)	19 (15.1)	0.43
Fluid intake, day 0 (ml)	2950 [1975–3060]	2825 [1975–3060]	0.74
Fluid intake, day 1 (ml)	2982 [2047–3945]	2948 [2080–3685]	0.71
Fluid intake, day 2 (ml)	2455 [1807–3756]	2648 [2010–3305]	0.99
Fluid intake, day 3 (ml)	2695 [1877–3678]	2340 [1643–3283]	0.34

Mann–Whitney Rank Sum test, *t*-test or chi2; *BE* base excess

### Urinary output

Fourteen of the 23 patients who developed CA-AKI (61 %) had altered urine output during the study period (1 “R”, 5 “I” and 8 “F” criteria from the RIFLE); in 10 of these 14 patients (70 %), the decreased urine output was noted before or at the same time as the increased serum creatinine concentrations.

Forty-five patients (30.2 %) had at least one RIFLE urine output criterion during the study period. Fourteen of these 45 developed CA-AKI, based on the creatinine criteria [18], associated with at least one RIFLE urine output criterion. Thus, 31 of these 45 patients had only RIFLE urine output criterion during the three days of the study period.

The sensitivity and specificity of the RIFLE urine output criteria as a screening test for CA-AKI as defined by Barrett and Parfrey [18], were 39.1 and 67.9 %, respectively, with a positive predictive value of 50.0 % and a negative predictive value of 87.2 %. The positive likelihood was 1.2 and the negative likelihood was 0.90.

We also compared the clinical, biological and haemodynamic data in patients with CA-AKI defined by Barrett and Parfrey (*n* = 9), those with CA-AKI and RIFLE urine output criteria (*n* = 14), those with RIFLE urine output criteria alone (*n* = 31), and those without changes in urine output or creatinine (*n* = 95) (Table 3). Patients with CA-AKI and RIFLE urine output criteria had a higher weight, a higher baseline creatinine concentration, need more RRT and higher mortality compared to patients without CA-AKI or RIFLE urine output criteria (Table 3). Despite higher doses of vasopressors, mean blood pressure was significantly lower with CA-AKI and RIFLE urine output criteria from 12 h post CM injection to days 3 compared to patients without CA-AKI and RIFLE urine output criteria (Table 3). Patients with only RIFLE criteria have the same length of ICU stay and ICU mortality than others but need more RRT than patients with CA-AKI based on creatinine levels, or without CA-AKI.

**Table 3:** Comparisons of clinical, biological and haemodynamic characteristics between patients who developed CA-AKI based on creatinine levels, CA-AKI with RIFLE urinary output criteria, with RIFLE urine output criteria only, or without CA-AKI or RIFLE urine output criteria

	CA-AKI and RIFLE (*n* = 14)	CA-AKI (*n* = 9)	Only RIFLE (*n* = 31)	No CA-AKI (*n* = 95)	*P* value
Age (years)	73 [61–80]	60 [48–67]	68 [57–75]	63 [55–71]	0.08
Sex (male/female)	10/4	6/3	22/9	55/40	0.55
ICU admission diagnosis (medical/surgical)	11/3*	7/2	29/2*	77/18	0.41
APACHE II score	26 [20–30]*	21 [17–28]	18 [11–26]	20 [14–23]	0.04
Weight (kg)	80 [74–90]	72 [60–88]	84 [75–95]*	75 [65–86]	0.05
Diabetes mellitus (%)	4 (29)	2 (22)	9 (29)	26 (27)	0.98
Nephrotoxic drugs	5 (36)	4 (44)	14 (45)	43 (45)	0.92
Cardiovascular risk factors (%)	11 (79)	6 (75)	23 (74)	65 (68)	0.86
Need for renal replacement (%)	3 (21)*	0 (0)	3 (10)*	0 (0)	<0.001
Length of ICU stay (days)	15 [12–18]	10 [6–19]	8 [5–21]	12 [8–23]	0.42
ICU mortality (%)	9 (64)*	3 (33)	9 (29)	14 (15)	<0.001
Haematocrit (%)	32 .2 [29.2–40.2]	28.5 [24.8–33.0]	32.1 [26.9–40.8]	30.6 [27.1–36.6]	0.37
Urea (mg/dL)	50 [42–57]	40 [31–54]	40 [32–60]	44 [32–58]	0.57
Creatinine (mg/dL)	1.18 [1.00–1.40]*	0.80 [0.45–1.10]	0.98 [0.74–1.18]	0.84 [0.62–1.08]	0.03
MDRD mL/min/1.73 m2	52 [46–71]	80 [66–173]	76 [52–92]	82 [59–128]	0.06
Patients with creatinine ≥1.5 mg/dL	1 (9 %)	1 (14 %)	4 (12.9 %)	9 (9.5 %)	0.93
pH	7.37 [7.30–7.42]	7.42 [7.27–7.48]	7.36 [7.30–7.42]	7.42 [7.33–7.46]	0.08
BE (meq/L)	−2 [−6–2]	1 [−3–7]	−1 [−4–0]	1 [−2–5]	0.08
HCO3 (mmoL/L)	22.9 [20.7–25.2]	25.0 [23.5–31.4]	24.0 [21.4–27.8]	25.7 [22.0–30.3]	0.29
Na (mEq/L)	140 [136–142]	138 [134–140]	139 [137–143]	140 [137–143]	0.19
CPK (UI/mL)	171 [91–854]	203 [11–376]	199 [49–604]	117 [44–348]	0.32
CRP (mg/dL)	15.6 [6.6–22.8]	8.7 [3.6–17.3]	7.4 [0.5–13.0]	9.3 [2.5–18.4]	0.13
Lactate (mmol/L)	1.0 [0.6–1.3]	0.6 [0.5–0.9]	0.4 [0.4–0.8]	0.5 [0.5–0.7]	0.11
Nephropathy prevention (%)	2 (14.2)	0 (0)	6 (19.4)	21 (22)	0.41
Mean blood pressure before CM (mmHg)	80 [64–94]	70 [62–87]	84 [74–91]	81 [71–94]	0.51
Mean blood pressure at 6 h (mmHg)	72 [66–88]	72 [60–79]	77 [68–88]	81 [71–96]	0.10
Mean blood pressure at 12 h (mmHg)	73 [64–74]*	74 [68–80]	74 [69–79]	79 [70–88]	0.02
Mean blood pressure at day 1 (mmHg)	71 [65–74]*	76 [69–80]	74 [68–83]	85 [74–97]	<0.001
Mean blood pressure at day 2 (mmHg)	67 [64–77]*	74 [68–77]*	79 [71–86]*	90 [79–98]	<0.001
Mean blood pressure at day 3 (mmHg)	76 [69–88]*	71 [65–85]*	77 [69–88]*	88 [77–100]	<0.001
CVP before CM (mmHg)	10 [6–14]	13 (11–14]	10 [8–11]	9 [5–11]	0.14
Maximal vasopressor dose on the day of CM (mcg/kg/min)	0.12 [0.00–0.20]* vs no	0.00 [0.00–0.15]	0.04 [0.00–0.12]	0.00 [0.00–0.00]	0.004
Maximal vasopressor dose, day 1 (mcg/kg/min)	0.10 [0.05–0.40]*	0.00 [0.00–0.06]	0.00 [0.00–0.06]	0.00 [0.00–0.00]	<0.001
Maximal vasopressor dose, day 2 (mcg/kg/min)	0.19 [0.00–0.40]*	0.00 [0.00–0.04]	0.00 [0.00–0.08]	0.00 [0.00–0.00]	<0.001
Maximal vasopressor dose, day 3 (mcg/kg/min)	0.15 [0.00–0.30]*	0.00 [0.00–0.09]	0.00 [0.00–0.01]	0.00 [0.00–0.00]	<0.001
Doses of CM (ml/kg)	1.54 [1.47–5.38]	1.64 [1.33–1.85]	1.53 [1.17–1.61]	1.60 [1.21–1.85]	0.32
Diuretics on day of CM injection (%)	1 (9 %)	5 (71 %)*	3 (12 %)	7 (13 %)	<0.001
Fluid intake, day 0 (ml)	3015 [2200–3885]	2495 [1774–3030]	2600 [2058–3516]	2830 [2124–3814]	0.65
Fluid intake, day 1 (ml)	3371 [2125–4184]	3371 [2125–4184]	3085 [2005–3748]	2865 [2098–3676]	0.49
Fluid intake, day 2 (ml)	3069 [1786–3780]	2090 [1730–3369]	2810 [2186–3124]	2550 [1949–3395]	0.78
Fluid intake, day 3 (ml)	2640 [1738–3811]	2750 [1820–3308]	2285 [1628–3103]	2465 [1690–3360]	0.63

Kruskal Wallis One way of variance on ranks with Dunn test correction

*BE* base excess

*p value <0.05 versus no CA-AKI

Finally, using a logistic regression analysis we assessed the prognostic parameters for development of CA-AKI and mortality (Tables 4 and 5). For the development of CA-AKI (Table 4), these factors were the APACHE II score, dose of CM injected by body weight, mean arterial blood pressure before CM injection, square root maximum dose of norepinephrine the day of CM injection, lactate and sodium concentrations the day of CM injection. For the mortality (Table 5), we included the same parameters plus the presence of CA-AKI. In the first model, only the square root of the dose of vasopressors before CM injection was significantly associated with the development of CA-AKI: OR 10.80 (1.87–62.34); *p* = 0.008 (Table 4). For the prediction of mortality, APACHE II score, dose of CM injected by body weight, CVP before CM injection, and presence of CA-AKI were significant (Table 5).

**Table 4:** Logistic regression to identify potential risk factors for development of CA-AKI

Risk factors	OR (IC 95 %)	*P* value
APACHE II score	1.02 (0.98–1.07)	0.33
Dose of CM injected by body weight	1.02 (0.80–1.30)	0.89
Na before CM	0.94 (0.88–1.01)	0.11
Mean arterial blood pressure before CM injection	1.00 (0.98–1.03)	0.81
Square root of the dose of vasopressors before CM injection	10.80 (1.87–62.34)	0.008
Lactate	1.00 (0.98–1.02)	0.99
CVP before CM injection	1.00 (1.00–1.01)	0.38

*OR* odds ratio and 95 % confidence intervals

*Na* sodium concentration

**Table 5:** Logistic regression to identify potential risk factors for mortality

Risk factors	OR (IC 95 %)	*P* value
APACHE II score	1.15 (1.07–1.23)	<0.0001
Presence of CA-AKI	0.25 (0.09–0.68)	0.007
Dose of CM	1.37 (1.00–1.86)	0.05
Na before CM	1.08 (0.97–1.20)	0.15
Mean arterial blood pressure before CM injection	1.00 (0.9−1.02)	0.29
Square root of the dose of vasopressors before CM injection	1.96 (0.28–13.64)	0.50
Lactate	1.01 (0.99–1.04)	0.24
CVP before CM injection	0.84 (0.71–0.94)	0.04

## Discussion

Our hypothesis was that urine output could be a predictive marker for detection of CA-AKI. To evaluate this possibility, we used the urine output criteria of the RIFLE classification [19]. We observed that the presence of at least one RIFLE urine output criterion had low sensitivity and specificity (39.1 and 67.9 %), a low positive predictive value of 50.0 % and a negative predictive value of 87.2 %. Only 57 % of the patients presented a “F” criteria of the RIFLE classification. Moreover, oliguria was observed prior to CA-AKI in only 9/23 patients (39 %). Thus, more than 60 % of the patients, oliguria developed when the creatinine concentration was already increasing, limiting the possibility of modifying therapy. Although oliguria may be an interesting marker of development of AKI in some situations [28], it is, therefore, probably of limited value in CA-AKI.

Despite the widespread use of CM injections in ICU patients, the epidemiology of CA-AKI is poorly described. In our retrospective study of 149 patients who were given a CM injection, we identified 23 patients (15.4 %) who developed CA-AKI during their ICU stay based on the definition of Barrett and Parfrey [18]. CA-AKI was associated with higher rates of renal replacement therapy (13 %) and increased ICU mortality (52 %). This frequency of CA-AKI is high, but in agreement with other recent studies. In a monocenter retrospective study over a 4.5 year period, Hoste et al. [15] reported that 16.3 % of the 787 patients receiving CM for CT scan or non-coronary angiography, developed CA-AKI based on creatinine concentrations [15]. Valette et al. [29] reported an incidence of 17 % in a population of surgical patients after CT scan, although 33 % of these patients had received another CM injection within a 72-h period, which may perhaps have resulted in an overestimation of the incidence of CA-AKI in this study [29]. For these reasons, we excluded patients who had a second CM injection. In contrast, Cely et al. identified only a 2 % occurrence of CA-AKI [14]. This large variation between studies is perhaps due to different definitions used for CA-AKI. Indeed, we used the same definition as Hoste et al. [15], based on the increase in serum creatinine of 25 % within 72 h after injection, whereas Cely et al. [14] used a decrease of 33 % of the measured creatinine clearance. Another difference with previous studies is the large number of patients in our study (34.2 %) who received CM for coronary angiography. Indeed, this population is more at risk of developing CA-AKI [16] for several reasons: First, the risk may be increased by the coronary procedure itself, as a result of low blood pressure and release of athero-embolic material during catheterization in the arterial circulation [16]. Moreover, the coronary procedure was performed after cardiac arrest in most cases, which may per se induce AKI, although this association remains controversial [30, 31]. Kidney function before CM injection (creatinine, metabolic acidosis) was more altered in our coronary angiography patients than in our CT patients; the coronary angiography patients were also more haemodynamically unstable during the survey period and received more CM/body weight. It thus seems logical to assume that an injection of CM could worsen the risk of CA-AKI by additional vasoconstriction and tubular injury.

We observed that mean blood pressure was lower at 12 h after CM injection and on day 2 and 3 in patients who developed CA-AKI compared to patients who did not. Nevertheless, mean blood pressure prior to CM injection was not significantly associated with CA-AKI in logistic regression. Our resuscitation process is based on recommendations for prevention of AKI in sepsis [5, 6, 32]. Indeed, Bourgoin et al. [5] observed no difference in diuresis, creatinine concentration and clearance in 28 patients with septic shock with a mean blood pressure of 65 mmHg compared to 85 mmHg [5]. Nevertheless, this study was performed over 8 h and no data at later timepoints (for example, 3 days) were reported. Albanese et al. [6] showed, in a prospective open label study, that norepinephrine increased urine output and decreased serum creatinine in septic but not in non-septic patients (head trauma with normal renal function) [6]. Moreover, it is possible that patients with chronic hypertension, as observed in 69.8 % of our population who developed CA-AKI, may need a higher mean blood pressure target [33]. Indeed, Asfar et al. [34] observed in a large septic population that higher levels of mean blood pressure (80–85 mmHg) was associated with less increase of creatinine levels and need for RRT in chronic hypertensive patients. Thus, the level of mean blood pressure was perhaps too low and may have contributed to the development of CA-AKI. This suggestion needs confirmation in prospective studies.

Our study has some limitations. First, the study was monocenter and retrospective. We have included only 3 % of the patients admitted in the ICU during the study period due to the strict inclusion and exclusion criteria. For these reasons, results should be taken with care to extra polate to other ICUs. Second, only 19.5 % of the patients who developed CA-AKI received preventive therapy. Indeed, a routine protocol was not applied to all patients in our ICU, as is proposed by the ESICM [35]; in contrast, only patients at risk of developing CA-AKI received preventive therapy. Discerning which patients are at risk can be difficult as shown by our data; moreover, urgent need for CT or angiography may limit the time available for applying a protocol, such as bicarbonate perfusion or hydration. Nevertheless, patients were already resuscitated before CM injection as suggested by the lactate concentrations, the mean arterial pressure and the CVP reported (Table 1).

Third, we investigated the potential deleterious effect of CM injection, but other factors may influence renal function in critical illness.

Fourth, as reported by several authors [15, 36, 37], studies reported the concept that AKI is not contrast-induced, and is an overstimated problem, because control patients without exposure to iodinated CM had a comparable incidence of AKI [36, 37].

## Conclusions

In conclusion, we have confirmed that CA-AKI is a frequent complication in ICU patients undergoing CT or coronary angiography and is associated with greater need for extra-renal epuration and increased mortality. The predictive value of the RIFLE urine output criteria for the development of CA-AKI diagnosed based on creatinine concentrations is low and limits their use for assessing the effects of therapeutic interventions on the development and progression of AKI.

## Abbreviations

AKI: acute kidney injury
APACHE II: Acute Physiology And Chronic Health Evaluation score
BE: base excess
CA-AKI: contrast-induced acute kidney injury
CM: contrast media
CT scan: computed tomography
CVP: central venous pressure
ICU: intensive care unit
MDRD: measured and estimated glomerular filtration rate
RIFLE: Risk of renal dysfunction, Injury to the kidney, Failure of kidney function, Loss of kidney function and End-stage renal disease
RRT: renal replacement therapy
